# Impact of Disease-Modifying Treatments on the Longitudinal Evolution of Anti-JCV Antibody Index in Multiple Sclerosis

**DOI:** 10.3389/fimmu.2018.02435

**Published:** 2018-10-25

**Authors:** Harald Hegen, Janette Walde, Gabriel Bsteh, Michael Auer, Sebastian Wurth, Anne Zinganell, Franziska Di Pauli, Florian Deisenhammer, Thomas Berger

**Affiliations:** ^1^Department of Neurology, Medical University of Innsbruck, Innsbruck, Austria; ^2^Department of Statistics, Faculty of Economics and Statistics, University of Innsbruck, Innsbruck, Austria

**Keywords:** JC virus, anti-JCV antibody index, natalizumab, interferon beta, glatiramer acetate, multiple sclerosis, seroconversion, longitudinal

## Abstract

**Background:** Risk of natalizumab-related progressive multifocal leukoencephalopathy is associated with the presence of anti-JC-virus (JCV) antibodies.

**Objective:** To investigate the impact of disease-modifying treatments (DMT) on the longitudinal evolution of anti-JCV antibody index.

**Methods:** Patients with multiple sclerosis who had serum sampling at intervals of 6 ± 3 months over up to 6 years and who either started DMT (interferon-β, glatiramer acetate or natalizumab) during the observation period with at least one serum sample available before and after treatment initiation or received no DMT during the observation period were included. Anti-JCV antibody serological status and index were determined by 2-step second-generation anti-JCV antibody assay.

**Results:** A total of 89 patients were followed for a median time of 55.2 months. Of those, 62 (69.7%) started DMT and 27 (30.3%) were without therapy during the observation period. Variation of longitudinal anti-JCV antibody index ranged from 9 to 15% and was similar in patients with and without DMT. Applying a mixed model considering the combined effects of treatment and time as well as individual heterogeneity did not show a significant change of anti-JCV antibody index by the start of treatment with interferon-β, glatiramer acetate, or natalizumab.

**Conclusion:** Evaluated DMTs do not impact longitudinal anti-JCV antibody index evolution.

## Introduction

Natalizumab (NTZ) treatment in multiple sclerosis (MS) patients is associated with the risk of progressive multifocal leukoencephalopathy (PML), an opportunistic infection of the brain caused by John Cunningham virus (JCV) (1). PML risk is determined by the prior use of immunosuppressants, duration of NTZ treatment and presence of serum anti-JCV antibodies (2). In seropositive patients, anti-JCV antibody index (AI) correlates with PML risk (3). In seronegative patients, seroconversion might occur with a rate of approximately 2–6% per year (4, 5). Previous studies evaluating the impact of DMT on anti-JCV antibodies yielded conflicting results, some of them claiming an increase of anti-JCV AI by NTZ treatment (6, 7).

Here, we aimed to investigate the impact of different DMTs on anti-JCV AI evolution in a cohort of MS patients using—in contrast to earlier studies—a longitudinal study design with high frequency sampling over a long observation time and with several samples available before and after start of the respective treatment.

## Methods

### Patients and samples

Out of a previously published cohort of MS patients who had serum sampling over 4–6 years at intervals of 6 ± 3 months (4), patients fulfilling one of the following (additional) criteria were included: (A) start with interferon-β (IFN-β) or glatiramer acetate (GLAT) therapy during the observation period with at least one serum sample available before and after treatment initiation (the sample immediately before treatment begin had to be off any prior treatment) *or* (B) start with NTZ therapy during the observation period with at least one serum sample available before and after treatment initiation, *or* (C) no DMT administration within the observation period. In groups A and B, all serum samples after treatment initiation were obtained while the patient was still on the same therapy.

### Anti-JCV antibody assay

Anti-JCV AI (and serological status) were determined at Unilabs (Copenhagen, Denmark) by a two-step enzyme-linked immunosorbent assay (STRATIFY JCV DxSelect; Focus Diagnostics, Cypress; CA, USA) as previously described (3, 8).

An anti-JCV AI >0.40 denoted anti-JCV antibody positivity and an index <0.20 denoted anti-JCV antibody negativity. For samples with an index ≥0.20 but ≤0.40 (intermediate response) further evaluation in the confirmation test was required. In the confirmation test, patient sample is pre-inhibited with the coating antigen in solution and, then, the pre-inhibited and non-inhibited aliquots of patient serum are tested. The results of the confirmation assay are reported as percentage inhibition, calculated as 100 × [1-(optical density of pre-inhibited/non-inhibited sample)]. Samples were scored eventually positive when inhibition was >45% (3, 8).

### Definition of seroconversion and seroreversion

Seroconversion was defined as occurrence of a positive anti-JCV antibody result at least once during follow-up, if baseline serostatus was negative. Seroreversion was defined as occurrence of a negative anti-JCV antibody testing at least once during the observation period in case of baseline positive serostatus. Hence, stable anti-JCV antibody status was defined by the same serological result obtained in all longitudinal samples per patient.

### Statistical analysis

Coefficient of variation (CV) of anti-JCV AI is displayed as the median of the CVs calculated for each patient by using all longitudinal anti-JCV AI. To test for statistical difference of the CV between each treatment group (IFN-β, GLAT, NTZ) and the no DMT group, a permutation test was applied for the median difference (10,000 runs).

**Figure 1:**
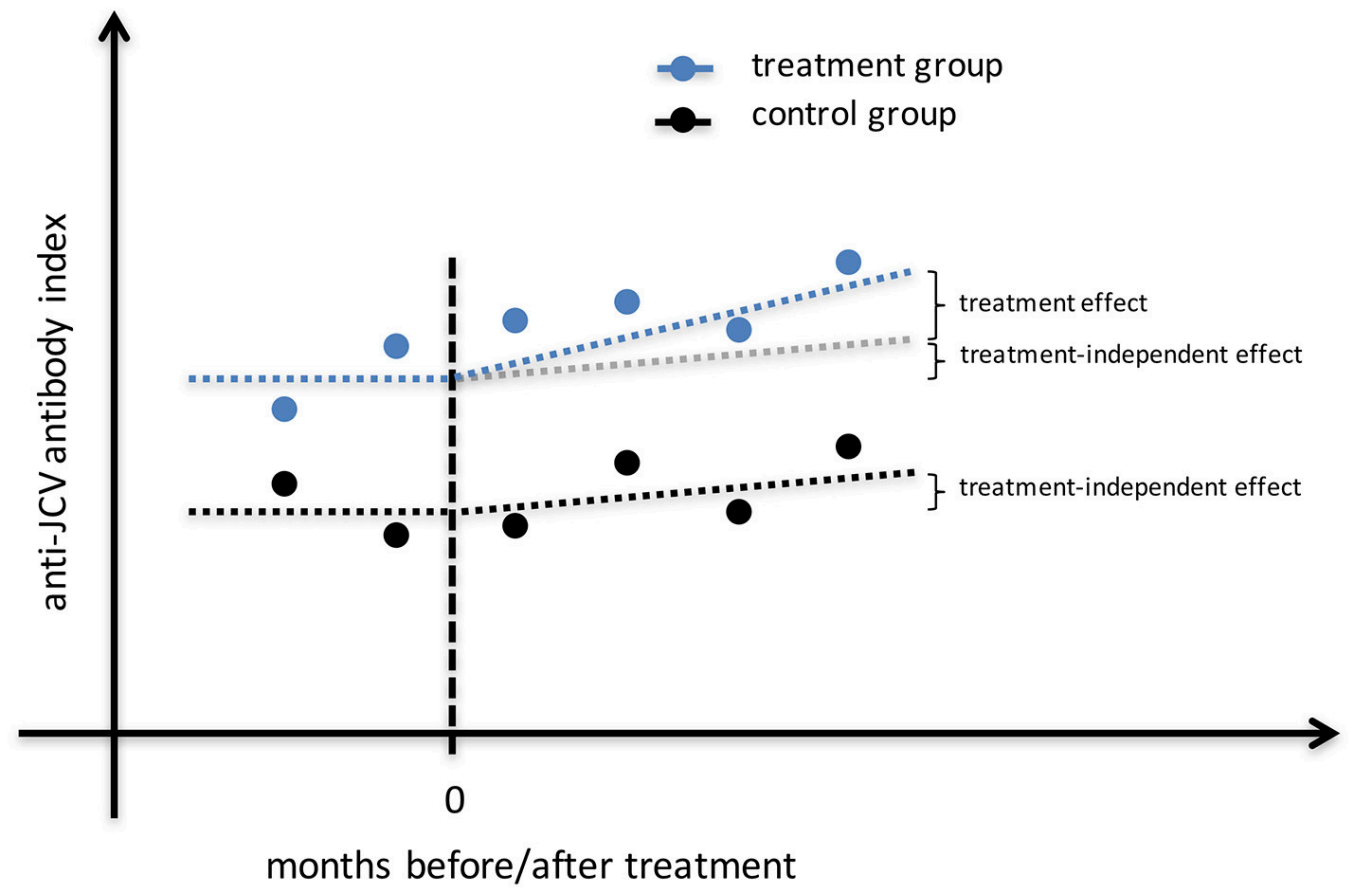
Study design for identification of treatment effect on anti-JCV antibody index. The illustrated estimation approach shows anti-JCV AI evolution for the no DMT group that may change (e.g., increase) over time, as well as anti-JCV AI evolution for a treatment group that may change (e.g., increase) due to the same effect as in the no DMT group plus a possible treatment effect. To correctly estimate the treatment effect, a mixed model is employed that considers any effect that appears also in the no DMT group independent of the applied treatment. Therefore, several samples before and after start of treatment per patient are required. AI, antibody index; DMT, disease modifying treatment; JCV, John Cunningham virus.

In order to investigate a possible increase of the anti-JCV AI after treatment a mixed model was employed (Figure 1). The variable *patient group* indicating the specific treatment (IFN-β, GLAT, NTZ, no DMT) and the variable *time* denoting two periods before treatment and four after treatment and their interaction were included in the regression equation. This time period was chosen as the dataset within these periods was almost balanced. The individual heterogeneity was modeled via the variables age, sex and random effects. Additionally, due to the time structure the within variance structure was assumed to follow an autoregressive process of order one. Furthermore, also an unstructured within-subject covariance was employed. Since the findings did not change quantitatively, we present the results of the approach with more degrees of freedom. Using joint tests the main effects (i.e., *patient group* and *time*) and the interaction effects were investigated. A power analysis was conducted regarding the combined effects of *time and patient group* considering repeated measurements and unequal sample sizes of employed patient groups (significance level = 5%, power = 80%, increase of anti-JCV antibody index after treatment = 0.2 per year).

*P* values were considered statistically significant at the level of 5%. Statistical analysis was done using Stata/MP 15.0 (StataCorp LLC, College Station, TX, USA). Permutation test and graphs were done in R system for statistical computing (9).

### Ethics

The study was approved by the ethics committee of Medical University of Innsbruck (approval number AN2014-0347 344/4.8). Written informed consent was obtained from all patients.

## Results

A total of 89 patients with a mean age of 36.6 years (SD 11.0) and a female predominance of 76.4% were included into the study, had median of 9 longitudinally collected serum samples and were followed for a median time of 55.2 months. Of those, 62 (69.7%) started DMT and 27 (30.3%) were without therapy during the observation period. None of the patients who started a DMT switched therapy and none of the untreated patients started any DMT during the observation period. Overall, 75 (84.3%) patients showed stable anti-JCV antibody status during the observation period.

### Longitudinal evolution of anti-JCV antibody index in untreated MS patients

**Figure 2:**
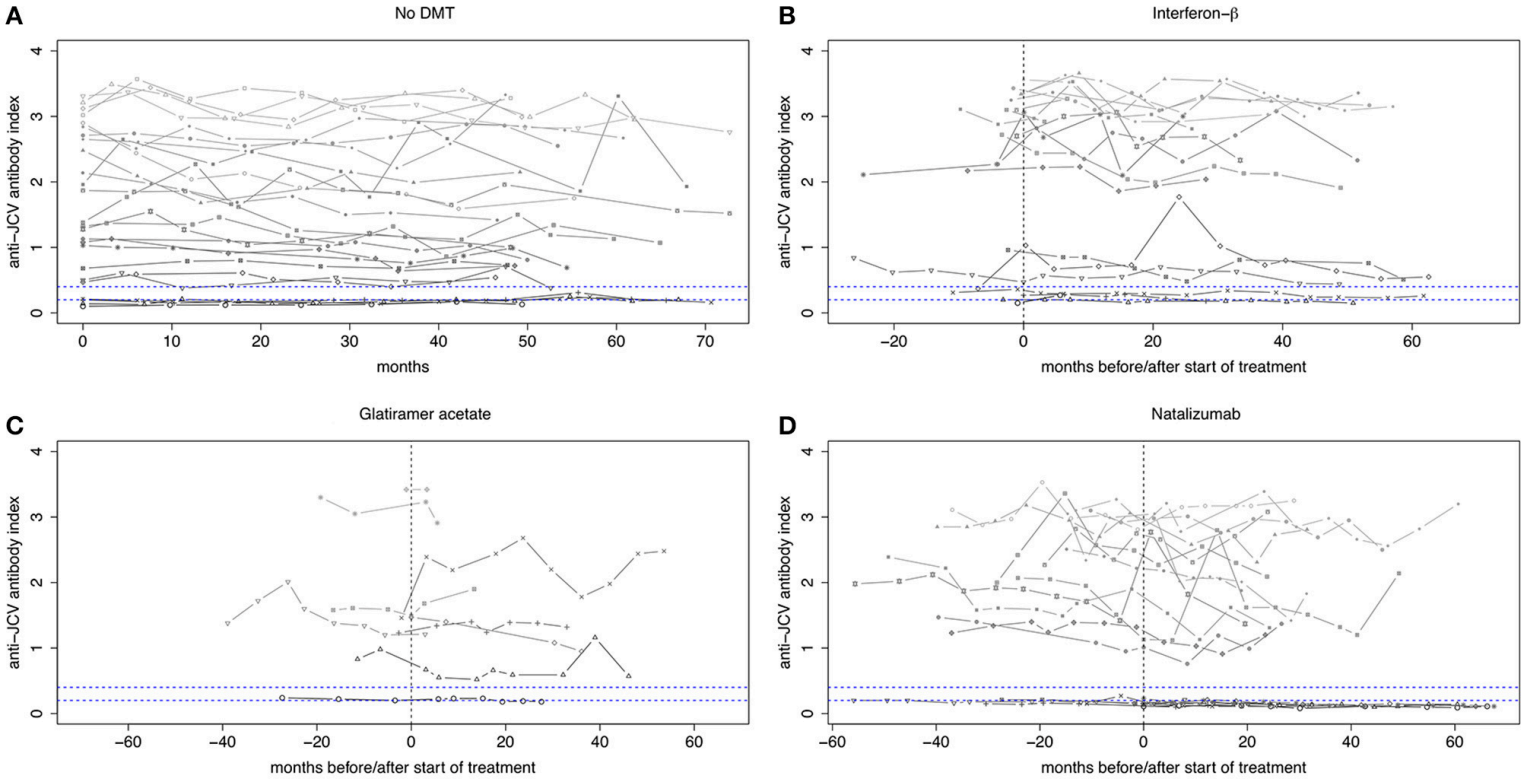
Longitudinal evolution of anti-JCV antibody index in patients with stable anti-JCV antibody status. **(A)** Serial anti-JCV antibody indices in patients without any disease-modifying treatment. **(B)** Serial anti-JCV antibody indices in patients before and after start of IFN-β therapy. Before, all patients were treatment-naïve. Afterwards, index values are shown as long as IFN-β was administered. **(C)** Serial anti-JCV antibody indices in patients before and after start of GLAT therapy. After treatment begin, index values are shown as long as GLAT was administered. **(D)** Serial anti-JCV antibody indices in patients before and after start of NTZ therapy. All but one patient received prior treatment. Afterwards, index values are shown as long as NTZ was applied. For building this graph, only patients with stable anti-JCV antibody status (i.e., without seroconversion/-reversion) during the observation period were included. Using a mixed model, there was no statistically significant change of anti-JCV antibody index before and after initiation of the respective treatment. *Vertical dashed line* indicates start of treatment. *Upper horizontal dashed line* indicates an anti-JCV antibody index of 0.4. Index values >0.4 are denoted anti-JCV antibody positive. *Lower horizontal dashed line* indicates an anti-JCV antibody index of 0.2. Index values <0.2 are denoted anti-JCV antibody negative. Samples with an index ≥0.20 but ≤0.40 (intermediate response) are classified as anti-JCV antibody positive or negative based on confirmation test (second step of the enzyme-linked immunosorbent assay), i.e., the displayed index values within this range might be classified as positive or negative. For further details see (3, 8). DMT, disease-modifying treatment; GLAT, glatiramer acetate; IFN-β, interferon-beta; JCV, John Cunningham virus; NTZ, natalizumab.

Twenty-seven patients without DMT were followed for median 53.9 months. Demographic and clinical data are shown in Table 1. Of those, 25 (92.6%) showed stable anti-JCV antibody status during the observation period. Anti-JCV AI did not significantly change over time neither including all patients (Table S1) nor patients with stable anti-JCV antibody status (Figure 2A, Table S2) or stable positive anti-JCV antibody status (Table S3). The median CV of anti-JCV AI in patients with stable anti-JCV antibody status was 14.4% (Table 2).

**Table 1:** Demographic and clinical characteristics of the study cohort.

	**No DMT**	**IFN-β**	**GLAT**	**NTZ**
Number of patients	27	25	9	28
Sex (female), *n* (%)	21 (77.8)	20 (80)	7 (77.8)	20 (71.4)
Age (years), mean (SD)	46.5 (10.9)	34.4 (8.5)	28.1 (8.6)	30.9 (7.1)
Disease duration (years), median (IQR)	9.0 (4.0–16.8)	3.6 (1.2–7.2)	4.9 (3.3–8.0)	5.4 (1.6–9.3)
Prior DMT, *n* (%)	11 (40.7)^a^	0	6 (66.7)^b^	27 (96.4)^c^
Time period between end of prior and begin of current DMT (months), median (IQR)	8.1 (2.7–89.4)^d^	n.a.	20.7 (13.8–48.2)	1.4 (0.7–2.3)
Number of longitudinal samples per patient, median	9	7	9	11
Observation period (months), median	53.9	49.3	65.1	64.2

a *Prior to the observation period, six patients received IFN-β, one GLAT and four patients immunosuppressive therapy (azathioprine and/ or cyclophosphamide)*.

b *Five patients were treated with IFN-β before starting GLAT therapy, one patient had already received GLAT once before*.

c *A total of 22 patients were on IFN-β and five patients on GLAT before switching to NTZ*.

d *In this patient group, time period between end of prior DMT and baseline visit is given*.

*DMT, disease-modifying treatment; GLAT, glatiramer acetate; IFN-β, interferon-β; IQR, interquartile range; n.a., not applicable; NTZ, natalizumab; SD, standard deviation*.

**Table 2:** Variability of longitudinal anti-JCV antibody index in patients with stable anti-JCV antibody status.

	**No DMT**	**IFN-β**	**GLAT**	**NTZ**
**ALL PATIENTS WITH STABLE ANTI-JCV ANTIBODY STATUS**				
CV of anti-JCV antibody index, (%) median (IQR)^a^	14.4 (7.2–19.7)	9.4 (7.0–16.3)	13.5 (5.7–18.8)	14.8 (9.8–21.0)
Anti-JCV antibody index, median	1.32	2.67	1.38	1.60
Number of patients	25	20	9	21
Number of longitudinal samples per patient, median	8	7.5	9	10
**PATIENTS WITH STABLE NEGATIVE ANTI-JCV ANTIBODY STATUS**				
CV of anti-JCV antibody index, (%) median (IQR)^a^	18.2 (16.6–19.7)	16.5 (13.0–23.9)	10.9	15.4 (14.2–20.6)
Anti-JCV antibody index, median	0.18	0.21	0.22	0.14
Number of patients	4	4	1	8
Number of longitudinal samples per patient, median	11.5	11.5	9	9.5
**PATIENTS WITH STABLE POSITIVE ANTI-JCV ANTIBODY STATUS**				
CV of anti-JCV antibody index, (%) median (IQR)^a^	12.1 (6.8–15.5)	7.6 (6.0–13.0)	15.1 (5.6–19.4)	10.9 (8.0–21.0)
Anti-JCV antibody index, median	1.85	2.84	1.48	2.18
Number of patients	21	16	8	13
Number of longitudinal samples per patient, median	8	7.5	8.5	10

a *Coefficient of variation (CV) is displayed as the median of the CVs calculated for each patient using all longitudinally determined anti-JCV antibody indices. Only patients with stable JCV serostatus during the observation period were included*.

*DMT, disease modifying treatment; GLAT, glatiramer acetate; IFN-β, interferon-β; JCV, John Cunningham virus; IQR, interquartile range; n.a., not applicable; NTZ, natalizumab*.

### Longitudinal evolution of anti-JCV antibody index before and after start of DMT

Out of 62 patients, who were followed for median 55.9 months, 25 (40.3%) started treatment with IFN-β, 9 (14.5%) with GLAT and 28 (45.2%) with NTZ. Demographic and clinical data are shown in Table 1.

### Interferon-beta

All patients starting IFN-β were treatment-naïve before. Twenty (80%) patients did not change their initial anti-JCV antibody status during the observation period. Using the mixed model, there was no statistically significant change of anti-JCV AI by the start of IFN-β therapy regardless of including all patients (Table S1), patients with stable anti-JCV antibody status (Figure 2B, Table S2) or stable positive anti-JCV antibody status (Table S3). Median CV of anti-JCV AI in patients with stable anti-JCV antibody status was 9.4% (Table 2) and did not statistically significantly differ from the no DMT group (*p* = 0.127).

### Glatiramer acetate

Three of nine (33.3%) patients starting GLAT were treatment-naïve before. In the remaining six patients, prior DMT was stopped median 20.7 months before. In all patients, at least the sample immediately before start of GLAT therapy was collected while being off any prior DMT. During the observation period, none of the patients showed seroconversion or seroreversion. As determined by the mixed model, there was no change of anti-JCV AI by the start of GLAT therapy including all (and therefore also serostable) patients (Figure 2C, Tables S1, S2) or patients with stable positive anti-JCV antibody status only (Table S3). Median CV of anti-JCV AI was 13.5% (Table 2) and did not significantly differ from the no DMT group (*p* = 0.449).

### Natalizumab

All but one patient, who switched to NTZ treatment during the observation period, received prior DMT and stopped it median 1.4 months before NTZ initiation. Twenty-one (75%) patients showed stable anti-JCV antibody status during follow-up. There was no statistically significant change in anti-JCV AI due to start of NTZ therapy including all patients (Table S1), patients with stable anti-JCV antibody status (Figure 2D, Table S2) or stable positive anti-JCV antibody status (Table S3). Median CV of anti-JCV AI in the serostable group was 14.8% (Table 2) and was similar as compared to the no DMT group (*p* = 0.699).

### Patients with seroconversion or seroreversion

**Figure 3:**
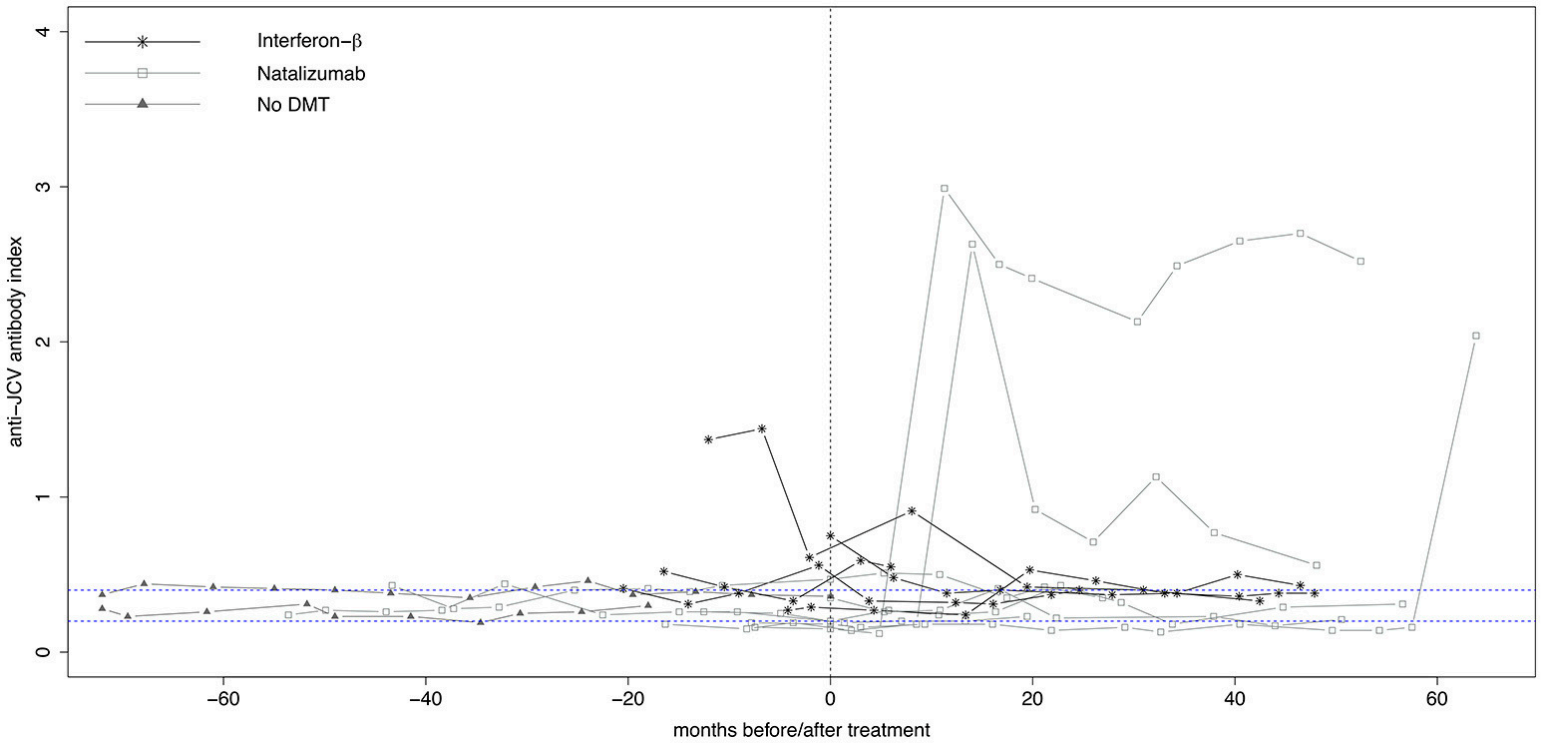
Longitudinal evolution of anti-JCV antibody index in patients with changing anti-JCV antibody status. Serial anti-JCV antibody indices in patients before and after start of interferon-β or natalizumab therapy, as well as in patients without DMT are shown. For building this graph, only patients with changing anti-JCV antibody status (i.e., with either seroconversion or seroreversion) during the observation period were included. Patients without DMT appear per definition left of the vertical dashed line, as in this group no treatment is commenced. *Vertical dashed line* indicates start of treatment. *Upper horizontal dashed line* indicates an anti-JCV antibody index of 0.4. Index values >0.4 are denoted anti-JCV antibody positive. *Lower horizontal dashed line* indicates an anti-JCV antibody index of 0.2. Index values <0.2 are denoted anti-JCV antibody negative. Samples with an index ≥0.20 but ≤0.40 (intermediate response) are classified as anti-JCV antibody positive or negative based on confirmation test (second step of the enzyme-linked immunosorbent assay), i.e., the displayed index values within this range might be classified as positive or negative. For further details see (3, 8). DMT, disease-modifying treatment; JCV, John Cunningham virus.

Longitudinal evolution of anti-JCV AI in those 14 (15.7%) patients who showed either seroconversion (*n* = 6, 42.9%) or seroreversion (*n* = 8, 57.1%) is shown in Figure 3. Five patients started treatment with IFN-β and seven with NTZ, while two patients where without any DMT during the observation period. Out of the 12 patients receiving treatment, seven patients changed anti-JCV antibody status while on treatment, the remaining five before treatment initiation.

## Discussion

Here we observed that evolution of anti-JCV AI is not influenced by the administration of DMT using—for the first time—a longitudinal study design with samples available before and after start of therapy.

To date, several studies have investigated the influence of different variables on serum anti-JCV antibodies. In this context, it has to be distinguished whether the influence of the variable of interest (e.g., age) on either anti-JCV antibody status or index was investigated, and it has to be distinguished whether a cross-sectional study design (establishing an association between the variable of interest and anti-JCV antibody status or index) or a longitudinal study design (assessing the change over time, i.e., seroconversion/-reversion or change in anti-JCV AI) was applied. By cross-sectional design, higher anti-JCV antibody prevalence (5, 10–17) and indices (4, 6) were observed with increasing patients' age, as well as in most studies higher antibody prevalence in males (5, 10–12, 14, 16, 18). Prior use of DMTs had no impact on anti-JCV antibody positivity (11–14, 16, 17) and index (10). By longitudinal design, age (4) and baseline anti-JCV AI (4, 19) were predictors of later anti-JCV antibody serostatus change, whereas no influence of prior and current DMTs on seroconversion rate were observed (14, 17). One study reported an increase in the annual rate of seroconversion with NTZ treatment duration, however, these higher rates were observed at the end of follow-up when the number of patients were small due to high drop-outs (e.g., only 20 of 85 patients remained in the study at year 5) (18). With respect to longitudinal anti-JCV AI evolution, two recent studies observed an increase of anti-JCV AI while on NTZ therapy (6, 7). Both studies compared anti-JCV AI of two consecutive samples that were both collected while patients received treatment with NTZ with a time period of approximately 1 year in between (6, 7). The first study found that the observed increase of anti-JCV AI (of ~0.1 per year) was higher than expected and explainable by the effect of age (7). However, the effect of age was estimated by correlation of anti-JCV AI and age at baseline, and then extrapolated over time. The second study focused on anti-JCV antibody positive patients and reported an increase of anti-JCV AI in this subgroup of patients. The authors argued that the increase of anti-JCV AI would go beyond an age effect, as there was no statistically significant correlation of age with anti-JCV AI in the anti-JCV antibody positive patient group (but in the whole cohort that also includes seroconverters) (6).

From a methodological point of view, the bivariate (cross-sectional) correlation between anti-JCV AI and baseline age cannot be estimated without bias (when no control variables are included or heterogeneity is not considered). Furthermore, it seems obvious that samples are needed before and after start of treatment to reliably assess the impact of treatment on anti-JCV AI evolution, and/ or to include a control group. Using a control allows the consideration of treatment-independent effects on anti-JCV AI evolution (Figure 1). Also, several sampling time points are required in order to minimize the possibility that a change in anti-JCV AI is artificially observed when comparing only two measurements against the background of a certain variability in anti-JCV AI over time.

Here, we applied a design that encounters the above-mentioned problems in assessing whether DMTs impact on anti-JCV AI. Even though the requirement of study design to include patients with long follow-up (median 5 years) and multiple consecutive samples (median 9 samples; available before as well as after start of therapy) resulted in a moderate total number of patients, especially compared to other previous studies, we still had a statistical power for our hypothesis (tested with the mixed model) of 80%. Power calculation was based on the decision to consider an increase in anti-JCV AI of 0.2 per year as relevant. This magnitude was based on our observation of the CV of anti-JCV AI over time, that in case of a statistically significant finding by the model, this would mean a true change in anti-JCV AI that goes beyond the “natural” fluctuation. Accordingly, the maximum increase of anti-JCV AI per year that has been reported by previous studies (6, 7) is within this variability, as e.g., a variability of 10% at an anti-JCV AI of 2.0 might result in an index of 2.2. Whereas reproducibility of the anti-JCV antibody assay has been shown to be high (6, 8), there has been so far no analysis of the “natural” long-term variation of anti-JCV AI over time [besides the longitudinal assessment of e.g., (bi-) annual anti-JCV antibody prevalence (4) and median anti-JCV AI (4, 16)].

There are some limitations of our study. First, we used a subgroup (*n* = 89) of a previously published cohort (*n* = 154) that was based on the availability of samples (before as well as after treatment initiation). Nevertheless, we are confident that the presented results are reliable, as the demographic characteristics (such as age and sex) as well as the clinical variables of interest (such as rate of seroconversion/ -reversion) are similar compared to the original cohort. Furthermore, the rate of seroconversion of ~3% per year is realistic (4). Higher conversion rates that were published by some prior studies [mostly between 10 to 15% per year, determined already within a relatively short observation period of approximately 1 year (3, 6, 10)] seem in our opinion somehow unrealistic, owing to the fact that anti-JCV antibody prevalence in MS patients is at least 50% (11), and applying these high seroconversion rates (of up to 15%) would implicate that after several years all patients have converted to anti-JCV antibody positivity. Secondly, to test our hypothesis we had a statistical power of 80%. Although this value is considered as a standard type II error and indeed a high number of samples were included, the number of patients was moderate. To further strengthen our findings, a higher statistical power (e.g., 90%) and thus a higher number of patients is desirable—an aim which has to be addressed by further studies. Another limitation of our study is that samples before start of NTZ were not treatment naïve, but on first-line treatment, in the majority of cases with IFN-β. This is because samples were collected during routine clinical visits and usually NTZ is used as second-line treatment. However, as IFN-β did not show an impact on anti-JCV AI over time (this group was therapy-naïve before), we hypothesize that pre-treatment with IFN-β will also have no impact on the analysis of NTZ samples. It seems indispensable that further studies should again not only address the impact of NTZ on longitudinal anti-JCV AI evolution, but also the impact of the various baseline DMT such as IFN-β so that the above drawn conclusion, that is starting of NTZ does not influence anti-JCV AI as assessed in pre-treated patients because the use of these pre-treatments has no impact on anti-JCV AI as well, can be confirmed.

## Author contributions

HH has participated in the conception and design of the study, acquisition, and statistical analysis of the data, and in drafting the manuscript. JW has participated in statistical analysis of the data and reviewing the manuscript for intellectual content. GB and MA has participated in data acquisition and reviewing the manuscript for intellectual content. SW, AZ, FDP, and FD have participated in reviewing the manuscript for intellectual content. TB has participated in the conception and design of the study and reviewing the manuscript for intellectual content.

### Conflict of interest statement

HH has participated in meetings sponsored by, received speaker honoraria or travel funding from Bayer, Biogen, Merck, Novartis, Sanofi-Genzyme and Teva, and received honoraria for acting as consultant for Teva. GB has participated in meetings sponsored by, received speaker honoraria or travel funding from Biogen, Merck, Novartis, Sanofi-Genzyme, and Teva, and received honoraria for acting as consultant for Teva. MA received speaker honoraria and/or travel funding from Biogen, Novartis, and Merck Serono. SW has participated in meetings sponsored by, received honoraria or travel funding from Biogen, Merck, Novartis, Sanofi-Genzyme, Teva Ratiopharm, Allergan, Ipsen Pharma, and Roche. FDP received travel funding and/or speaker honoraria from Biogen Idec and Sanofi-Genzyme. FD has participated in meetings sponsored by or received honoraria for acting as an advisor/speaker for Bayer, Biogen, Merck, Novartis, Roche, Sanofi-Genzyme, and Teva-Ratiopharm. His institution has received financial support for participation in randomized controlled trials of INFb-1b (Betaferon, Bayer Schering Pharma), INFb-1a (Avonex, Biogen; Rebif, Merck Serono), glatiramer acetate (Copaxone, Teva Pharmaceuticals), Natalizumab (Tysabri, Biogen), in multiple sclerosis. He is section editor of the MSARD Journal (Multiple Sclerosis and Related Disorders). TB has participated as a consultant in meetings sponsored by and received honoraria (lectures, advisory boards, consultations) in the past 12 months from pharmaceutical companies marketing treatments for multiple sclerosis: Biogen, Bionorica, Celgene, MedDay, Merck, Novartis, Roche, Sanofi-Genzyme, and TEVA ratiopharm. TB and his institution have received financial support by unrestricted research grants and clinical trial participation from Alexion, Bayer, Biogen, Merck, Novartis, Roche, Sanofi-Genzyme, and TEVA ratiopharm. The remaining authors declare that the research was conducted in the absence of any commercial or financial relationships that could be construed as a potential conflict of interest.

## References

[1] Tan CS Koralnik IJ. Progressive multifocal leukoencephalopathy and other disorders caused by JC virus: clinical features and pathogenesis. Lancet Neurol. (2010) 9:425–37. 10.1016/S1474-4422(10)70040-5 20298966 PMC2880524

[2] Bloomgren G Richman S Hotermans C Subramanyam M Goelz S Natarajan A. Risk of natalizumab-associated progressive multifocal leukoencephalopathy. N Engl J Med. (2012) 366:1870–80. 10.1056/NEJMoa1107829 22591293

[3] Plavina T Subramanyam M Bloomgren G Richman S Pace A Lee S. Anti-JC virus antibody levels in serum or plasma further define risk of natalizumab-associated progressive multifocal leukoencephalopathy. Ann Neurol. (2014) 76:802–12. 10.1002/ana.24286 25273271 PMC4282070

[4] Hegen H Auer M Bsteh G Di Pauli F Plavina T Walde J Deisenhammer F Berger T. Stability and predictive value of anti-JCV antibody index in multiple sclerosis: a 6-year longitudinal study. PLoS ONE (2017) 12:e0174005. 10.1371/journal.pone.0174005 28319193 PMC5358769

[5] Gorelik L Lerner M Bixler S Crossman M Schlain B Simon K. Anti-JC virus antibodies: implications for PML Risk Stratification. Ann Neurol. (2010) 68:295–303. 10.1002/ana.22128 20737510

[6] Schwab N Schneider-Hohendorf T Pignolet B Breuer J Gross CC Göbel K. Therapy with natalizumab is associated with high JCV seroconversion and rising JCV index values. Neurology Neuroimmunol Neuroinflam. (2016) 3:e195. 10.1212/NXI.0000000000000195 26848486 PMC4733149

[7] Raffel J Gafson AR Malik O Nicholas R. Anti-JC virus antibody titres increase over time with natalizumab treatment. Multiple Scler. (2015) 21:1833–8. 10.1177/1352458515599681 26449743

[8] Lee P Plavina T Castro A Berman M Jaiswal D Rivas S. A second-generation ELISA (STRATIFY JCV™ DxSelect™) for detection of JC virus antibodies in human serum and plasma to support progressive multifocal leukoencephalopathy risk stratification. J Clin Virol. (2013) 57:141–6. 10.1016/j.jcv.2013.02.002 23465394

[9] R Core Team R: A Language and Environment for Statistical Computing. Available online at: www.R-project.org

[10] Trampe AK Hemmelmann C Stroet A Haghikia A Hellwig K Wiendl H. Anti-JC virus antibodies in a large German natalizumab-treated multiple sclerosis cohort. Neurology (2012) 78:1736–42. 10.1212/WNL.0b013e3182583022 22592369

[11] Olsson T Achiron A Alfredsson L Berger T Brassat D Chan A. Anti-JC virus antibody prevalence in a multinational multiple sclerosis cohort. Mult Scler. (2013) 19:1533–8. 10.1177/1352458513477925 23459571

[12] Bozic C Richman S Plavina T Natarajan A Scanlon JV Subramanyam M. Anti-John Cunnigham virus antibody prevalence in multiple sclerosis patients: baseline results of STRATIFY-1. Ann Neurol. (2011) 70:742–50. 10.1002/ana.22606 22162056

[13] Salmen A Ahsen von N Trampe AK Hoepner R Plavina T Subramanyam M. Longitudinal analyses of anti-JCV antibody index for risk assessment of progressive multifocal leukoencephalopathy. Multiple Scler. J. (2016) 2:2055217316630008. 10.1177/2055217316630008 28607714 PMC5433507

[14] Alroughani R Akhtar S Ahmed SF Khoury SJ Al-Hashel JY Sahraian MA. JC virus seroprevalence and seroconversion in multiple sclerosis cohort: a Middle-Eastern study. J Neurol Sci. (2016) 360:61–5. 10.1016/j.jns.2015.11.044 26723975

[15] Aladro Y Terrero R Cerezo M Ginestal R Ayuso L Meca-Lallana V. Anti-JC virus seroprevalence in a Spanish multiple sclerosis cohort. J Neurol Sci. (2016) 365:16–21. 10.1016/j.jns.2016.03.050 27206867

[16] Kolasa M Hagman S Verkkoniemi-Ahola A Airas L Koivisto K Elovaara I. Anti-JC virus seroprevalence in a Finnish MS cohort. Acta Neurol Scand. (2016) 133:391–7. 10.1111/ane.12475 26347001

[17] Dominguez-Mozo MI Rus M Santiago JL Izquierdo G Casanova I Galan V. Study of the anti-JCV antibody levels in a Spanish multiple sclerosis cohort. Eur J Clin Invest. (2017) 47:158–66. 10.1111/eci.12721 28036121

[18] Correia I Jesus-Ribeiro J Batista S Martins AI Nunes C Macário MC. Anti-JCV antibody serostatus and longitudinal evaluation in a Portuguese Multiple Sclerosis population. J Clin Neurosci. (2017) 45:257–60. 10.1016/j.jocn.2017.08.006 28844615

[19] Vennegoor A van Rossum JA Leurs C Wattjes MP Rispens T Murk JLAN. High cumulative JC virus seroconversion rate during long-term use of natalizumab. Eur J Neurol. (2016) 23:1079–85. 10.1111/ene.12988 27018481

