# Supplementary material for: Impact of Disease-Modifying Treatments on the Longitudinal Evolution of Anti-JCV Antibody Index in Multiple Sclerosis
Source: Front Immunol. 2018 Oct 25;9:2435. doi: 10.3389/fimmu.2018.02435 (PMC6209669; doi:10.3389/fimmu.2018.02435)
Supplement: Supplementary file 1 [file Data_Sheet_1.PDF]

Table S1: Results of regression analysis including all patients

Effect

	df	Chi-square	p-value
Anti-JCV antibody index			
Patient group	3	3.92	0.271
Time	5	4.37	0.498
Patient group # Time	15	8.16	0.917

Mixed-effects REML regression

Number of observations = 444

Number of groups = 88

Anti-JCV antibody index	Coefficient	Stand. Error	z	P>z	95% confidence interval	
Difference of anti-JCV antibody index levels between patient groups						
Patient group (reference: 0 = no DMT)						
1 = IFN-β	0.678	0.382	1.77	0.076	-0.071	1.427
2 = GLAT	0.214	0.508	0.42	0.673	-0.781	1.210
3 = NTZ	0.045	0.374	0.12	0.904	-0.689	0.779
Change of anti-JCV antibody index over time						
Time (reference: 1 = -12 months before treatment start)						
2 = -6 months	-0.072	0.079	-0.91	0.364	-0.226	0.083
3 = first time on treatment	-0.009	0.102	-0.09	0.928	-0.210	0.192
4 = 6 months	-0.078	0.122	-0.64	0.524	-0.317	0.161
5 = 12 months	-0.123	0.140	-0.88	0.380	-0.398	0.152
6 = 18 months	-0.077	0.159	-0.49	0.627	-0.388	0.234
Effect of treatment on longitudinal anti-JCV antibody index evolution						
Patient group # Time						
1 2	-0.071	0.151	-0.47	0.639	-0.366	0.224
1 3	-0.045	0.177	-0.26	0.797	-0.392	0.301
1 4	-0.087	0.199	-0.44	0.663	-0.477	0.303
1 5	-0.063	0.220	-0.29	0.773	-0.494	0.367
1 6	-0.188	0.240	-0.78	0.434	-0.658	0.283
2 2	0.124	0.175	0.71	0.478	-0.219	0.467
2 3	0.026	0.230	0.11	0.910	-0.424	0.476
2 4	-0.087	0.273	-0.32	0.749	-0.622	0.448
2 5	0.051	0.311	0.16	0.869	-0.559	0.661
2 6	0.008	0.345	0.02	0.981	-0.667	0.684
3 2	0.044	0.110	0.40	0.687	-0.171	0.260
3 3	-0.003	0.143	-0.02	0.981	-0.285	0.278
3 4	0.097	0.169	0.58	0.565	-0.234	0.428
3 5	0.201	0.193	1.04	0.296	-0.177	0.579
3 6	0.061	0.214	0.29	0.775	-0.359	0.482
Age	0.023	0.013	1.80	0.073	-0.002	0.049
Sex	-0.098	0.275	-0.36	0.721	-0.636	0.440
Constant	0.488	0.772	0.63	0.527	-1.026	2.002

Table S2: Results of regression analysis including patients with stable anti-JCV antibody status

Effect

	df	Chi-square	p-value
Anti-JCV antibody index			
Patient group	3	4.98	0.173
Time	5	7.71	0.173
Patient group # Time	15	4.07	0.998

Mixed-effects REML regression

Number of observations	=	363
Number of groups	=	74

Anti-JCV antibody index	Coefficient	Stand. Error	z	P>z	95% confidence interval	
Difference of anti-JCV antibody index levels between patient groups						
Patient group (reference: 0 = no DMT)						
1 = IFN-β	0.794	0.403	1.97	0.049	0.004	1.583
2 = GLAT	0.165	0.508	0.32	0.745	-0.831	1.161
3 = NTZ	0.280	0.395	0.71	0.478	-0.494	1.054
Change of anti-JCV antibody index over time						
Time (reference: 1 = -12 months before treatment start)						
2 = -6 months	-0.075	0.069	-1.08	0.278	-0.209	0.060
3 = first time on treatment	-0.003	0.089	-0.03	0.972	-0.178	0.172
4 = 6 months	-0.078	0.106	-0.74	0.462	-0.287	0.130
5 = 12 months	-0.135	0.123	-1.10	0.273	-0.376	0.106
6 = 18 months	-0.072	0.140	-0.51	0.610	-0.346	0.203
Effect of treatment on longitudinal anti-JCV antibody index evolution						
Patient group # Time						
1 2	0.011	0.171	0.06	0.951	-0.325	0.347
1 3	0.034	0.189	0.18	0.859	-0.338	0.405
1 4	-0.015	0.205	-0.07	0.943	-0.417	0.388
1 5	0.028	0.222	0.13	0.899	-0.407	0.464
1 6	-0.132	0.239	-0.55	0.580	-0.600	0.336
2 2	0.089	0.159	0.56	0.576	-0.223	0.402
2 3	0.085	0.198	0.43	0.668	-0.304	0.474
2 4	0.121	0.241	0.50	0.616	-0.351	0.592
2 5	0.099	0.277	0.36	0.721	-0.443	0.641
2 6	0.049	0.308	0.16	0.874	-0.556	0.653
3 2	0.046	0.101	0.46	0.644	-0.151	0.244
3 3	0.011	0.132	0.08	0.935	-0.248	0.270
3 4	-0.015	0.155	-0.10	0.924	-0.319	0.289
3 5	0.013	0.178	0.08	0.940	-0.335	0.362
3 6	-0.073	0.198	-0.37	0.712	-0.461	0.315
Age	0.025	0.014	1.79	0.073	-0.002	0.052
Sex	-0.245	0.290	-0.85	0.398	-0.814	0.323
Constant	0.711	0.812	0.88	0.381	-0.881	2.302

Table S3: Results of regression analysis including patients with stable positive anti-JCV antibody status

Effect

	df	Chi-square	p-value
Anti-JCV antibody index			
Patient group	3	7.69	0.053
Time	5	8.45	0.1333
Patient group # Time	15	5.75	0.9837

Mixed-effects REML regression

Number of observations = 283

Number of groups = 57

Anti-JCV antibody index	Coefficient	Stand. Error	z	P>z	95% confidence interval	
Difference of anti-JCV antibody index levels between patient groups						
Patient group (reference: 0 = no DMT)						
1 = IFN-β	0.643	0.387	1.66	0.097	-0.116	1.401
2 = GLAT	-0.345	0.444	-0.78	0.437	-1.216	0.525
3 = NTZ	0.394	0.376	1.05	0.294	-0.342	1.130
Change of anti-JCV antibody index over time						
Time (reference: 1 = -12 months before treatment start)						
2 = -6 months	-0.083	0.087	-0.96	0.339	-0.254	0.087
3 = first time on treatment	0.016	0.111	0.15	0.882	-0.202	0.235
4 = 6 months	-0.061	0.131	-0.47	0.641	-0.317	0.195
5 = 12 months	-0.107	0.149	-0.72	0.472	-0.398	0.184
6 = 18 months	-0.019	0.171	-0.11	0.913	-0.353	0.316
Effect of treatment on longitudinal anti-JCV antibody index evolution						
Patient group # Time						
1 2	0.000	0.229	0.00	0.999	-0.449	0.450
1 3	0.037	0.249	0.15	0.883	-0.452	0.526
1 4	-0.018	0.266	-0.07	0.945	-0.540	0.503
1 5	0.030	0.285	0.11	0.916	-0.528	0.588
1 6	-0.162	0.304	-0.53	0.595	-0.758	0.435
2 2	0.152	0.170	0.89	0.372	-0.182	0.486
2 3	0.015	0.224	0.07	0.947	-0.425	0.455
2 4	-0.101	0.273	-0.37	0.712	-0.636	0.434
2 5	0.065	0.312	0.21	0.835	-0.546	0.676
2 6	-0.011	0.347	-0.03	0.976	-0.690	0.669
3 2	0.068	0.129	0.53	0.598	-0.184	0.320
3 3	0.051	0.173	0.29	0.770	-0.289	0.390
3 4	-0.009	0.203	-0.04	0.965	-0.407	0.390
3 5	0.015	0.233	0.06	0.948	-0.442	0.472
3 6	-0.084	0.259	-0.32	0.747	-0.591	0.424
Age	-0.003	0.013	-0.21	0.830	-0.028	0.023
Sex	-0.434	0.264	-1.65	0.100	-0.951	0.082
Constant	2.545	0.793	3.21	0.001	0.991	4.099
